# Supplementary material for: Solid-state NMR spectroscopy based atomistic view of a membrane protein unfolding pathway
Source: Nat Commun. 2019 Aug 27;10:3867. doi: 10.1038/s41467-019-11849-8 (PMC6711998; doi:10.1038/s41467-019-11849-8)
Supplement: Supplementary file 1 — Supplementary Information [file 41467_2019_11849_MOESM1_ESM.pdf]

Supplementary Information

Solid-state NMR spectroscopy based atomistic view of a membrane protein unfolding pathway

P. Xiao *et al.*

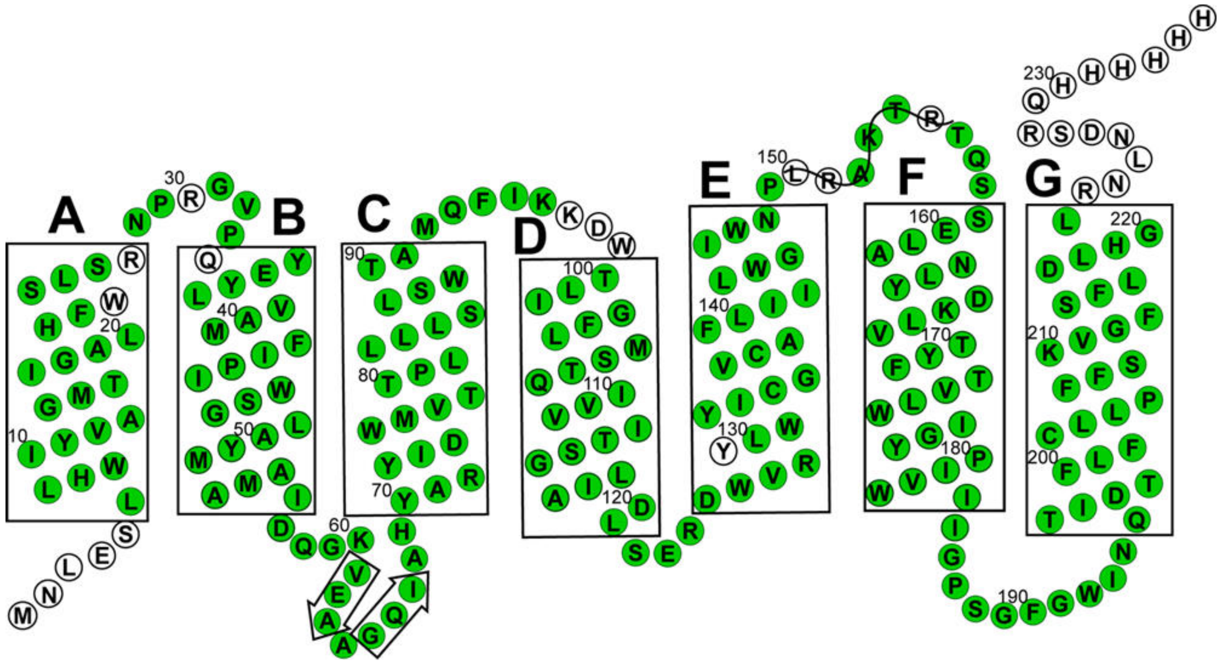

Supplementary Figure 1. The amino acid sequence, topology, and spectroscopic assignments of ASR. Transmembrane helices are represented as rectangles with cytoplasmic side on top, assigned residues are shown in green; BMRB entry: 18595. Reprinted with permission from D. Good, *et al.*, *J. Am. Chem. Soc.* **2014**, 136, 2833. Copyright 2014, American Chemical Society.

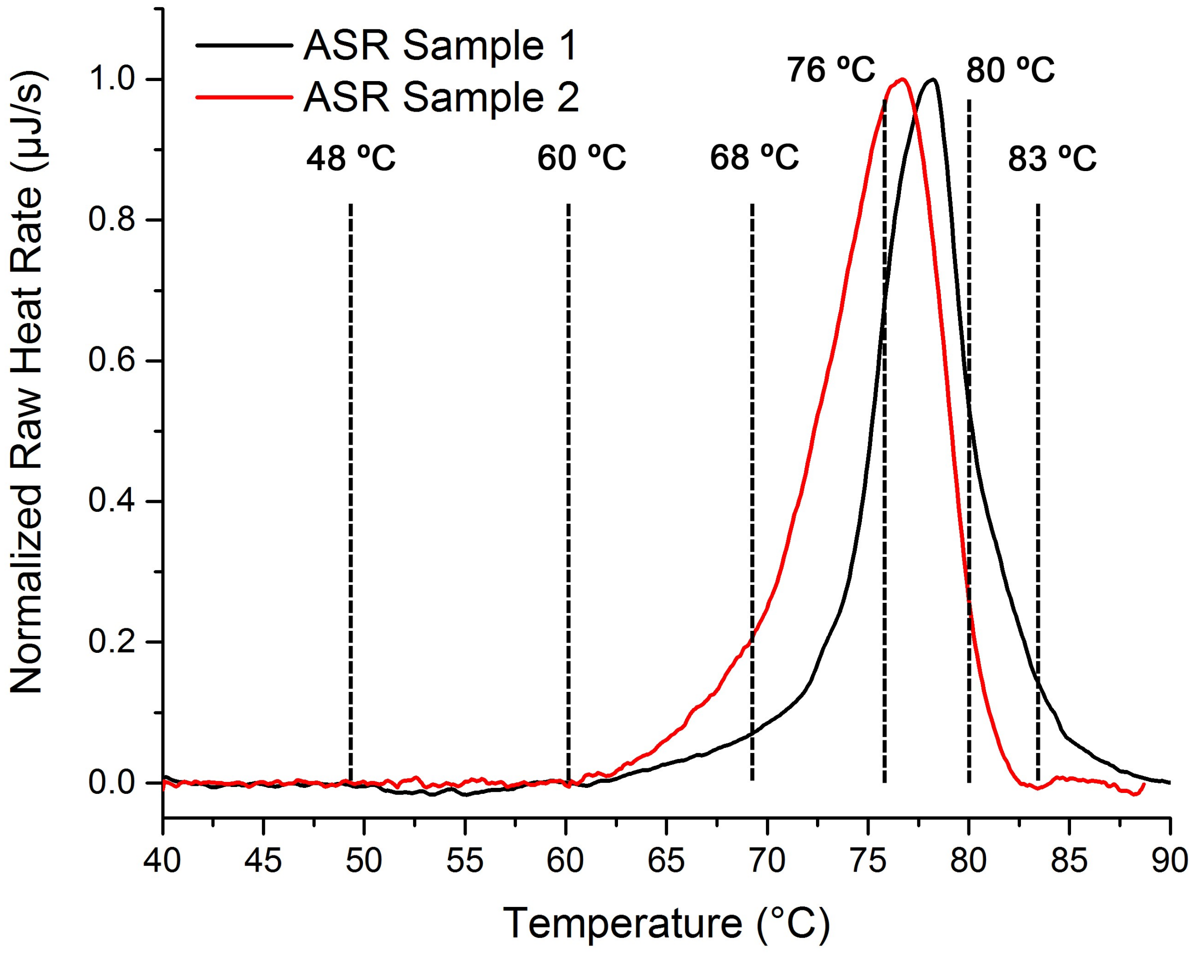

Supplementary Figure 2. DSC measurements on ASR proteoliposomes in D_2O buffer. Both DSC curves are presented after baseline correction and normalized by dividing maximum. Small differences between the DSC curves collected on two samples are related to small variation in protein-to-lipid ratios. Vertical dashed lines indicate approximate incubation temperatures for each NMR data set. Additional details are given in the Methods.

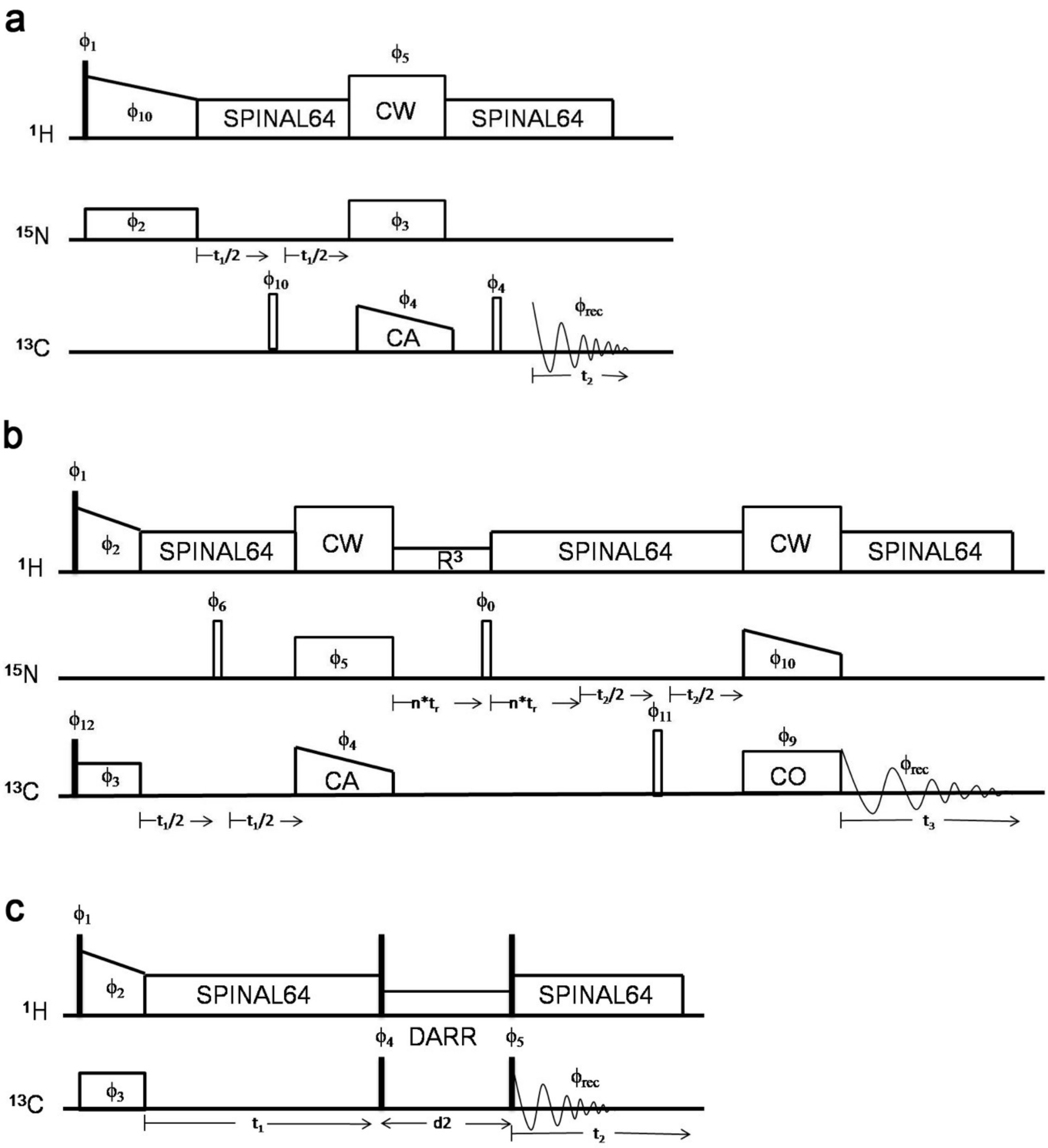

Supplementary Figure 3. NMR pulse sequences. (**a**) 2D NCA, (**b**) 3D R^3-CANCO, and (**c**) 2D ^{13}C -^{13}C with DARR mixing. Solid and hollow rectangles represent $\pi/2$ and π pulses, respectively. Phase tables were as follows: (**a**) φ_1 = (y, -y), φ_2 = (x), φ_3 = (x, x), φ_4 = (x, x, y, y, -x, -x, -y, -y), φ_5 = (x, -x), φ_{yx} = (x), φ_{rec} = (x, -x, y, -y, -x, x, -y, y). (**b**) φ_0 = (x), φ_1 = (y), φ_2 = (x, -x), φ_3 = (x), φ_4 = (x, x, x, x, -x, -x, -x, -x), φ_5 =(x, x, -x, -x), φ_6 = (x), φ_9 = (x, x, x, x, x ,x ,x, x, y, y, y, y, y, y, y, y), φ_{10} = (x), φ_{11} = (x),φ_{12} = (y, -y), φ_{rec} = (x, -x, -x, x, -x, x, x, -x, y, -y, -y, y, -y, y, y, -y). (**c**) φ_1 = (y, -y, y, -y), φ_2 = (x), φ_3 = (x, x, x, x, y, y, y, y -x, -x, -x, -x, -y, -y, -y, -y), φ_4 = (y, y, -y, -y, -x, -x, x, x, -y, -y, y, y, x, x, -x, -x), φ_5 = (y, y, y, y, -x, -x, -x, -x, -y, -y, -y, -y, x, x, x, x), φ_{rec} = (x, -x, -x, x, y, -y, -y, y, -x, x, x, -x, -y, y, y, -y).

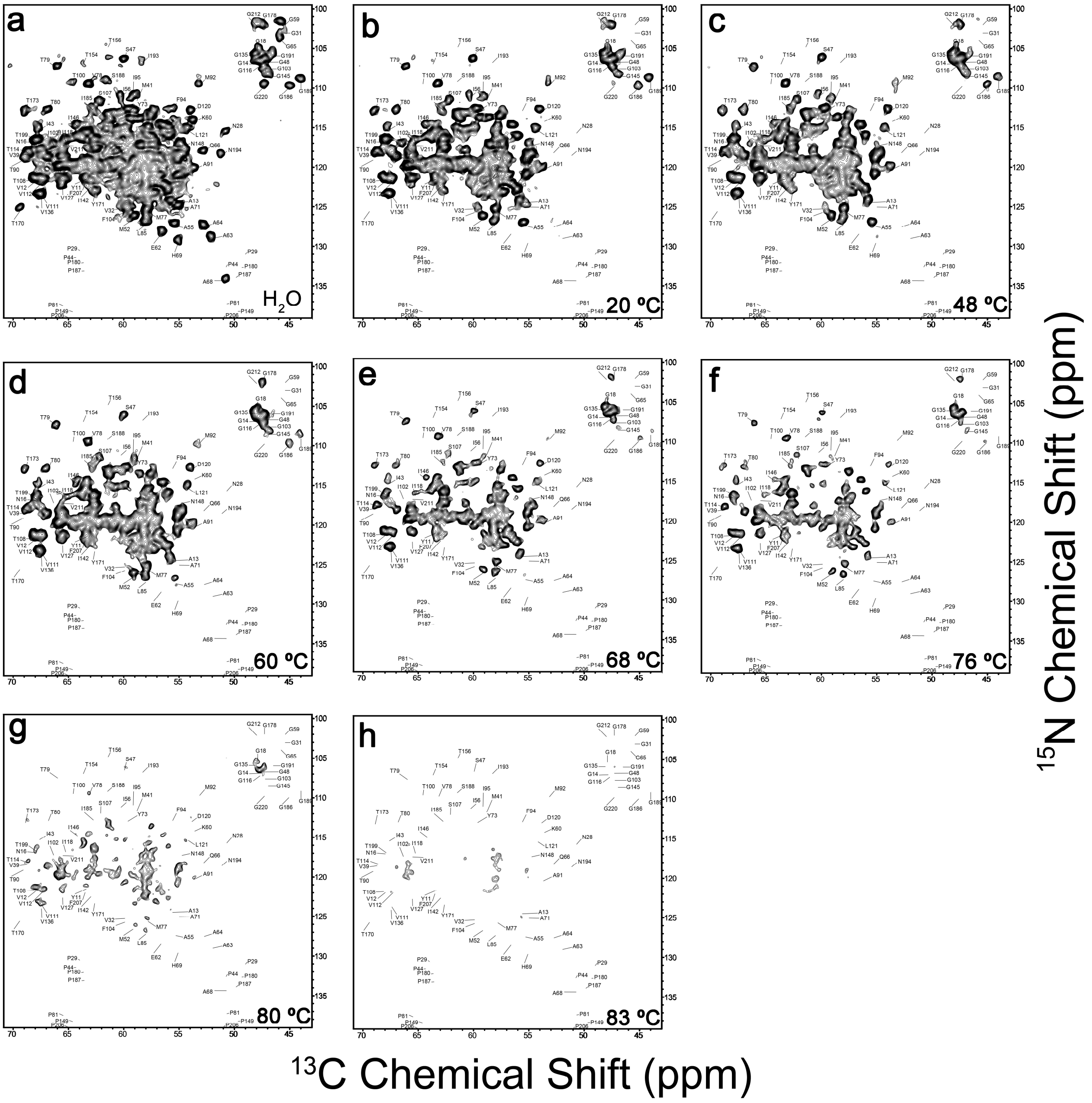

Supplementary Figure 4. A series of 2D NCA correlation spectra as a function of incubation temperature. (**a**) Reference 2D NCA spectrum collected in the H_2O based buffer at 20 °C. (**b** to **h**) 2D NCA spectra collected after incubation in the D_2O based buffer at indicated temperatures. All spectra were collected on sample 1. The first contour is at 5 times root-mean-square of the noise.

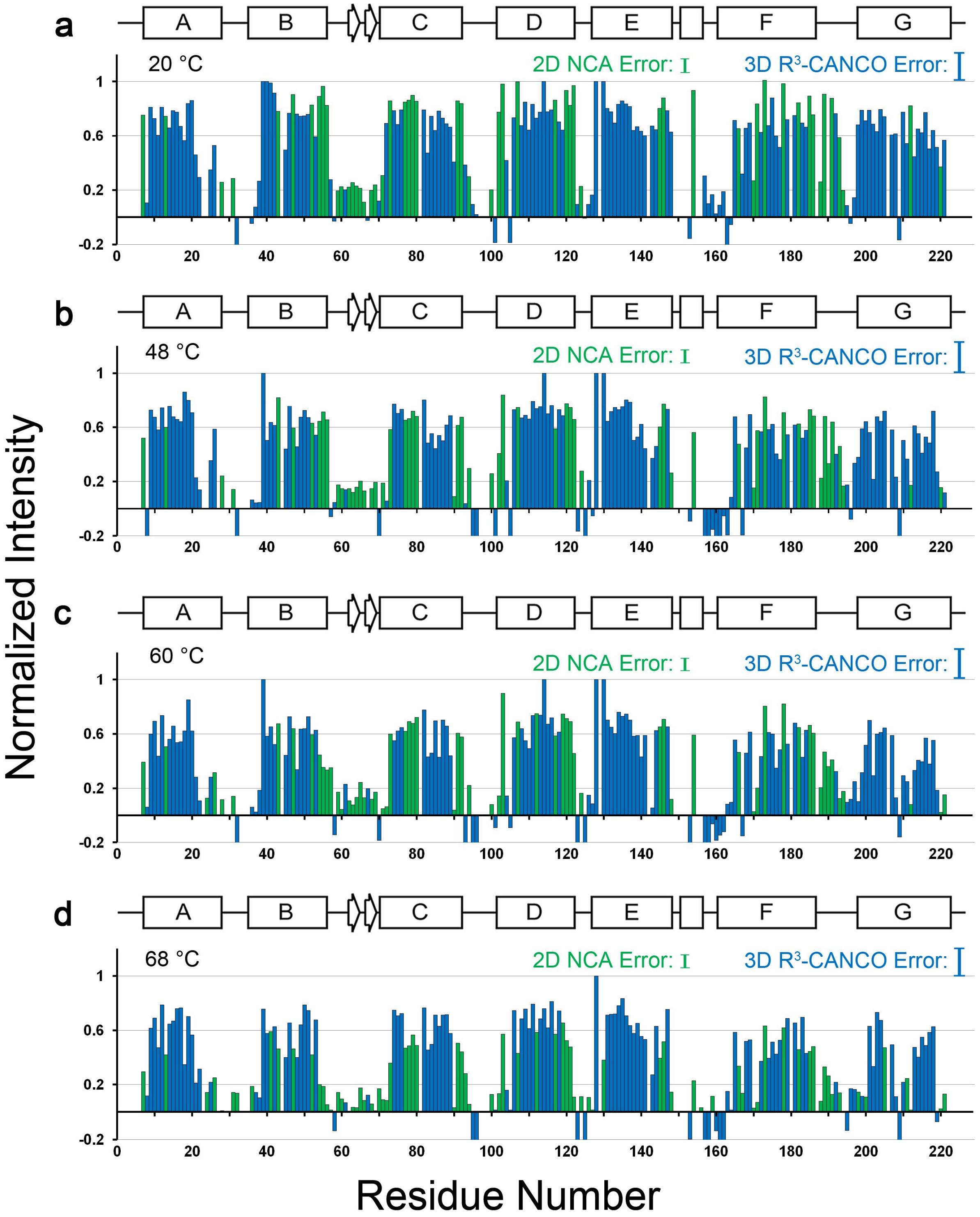
a
b
c
d
A
B
C
D
E
F
G
20 °C
48 °C
60 °C
68 °C
2D NCA Error:
3D R³-CANCO Error:
Normalized Intensity
Residue Number
1
0.6
0.2
-0.2
0
20
40
60
80
100
120
140
160
180
200
220

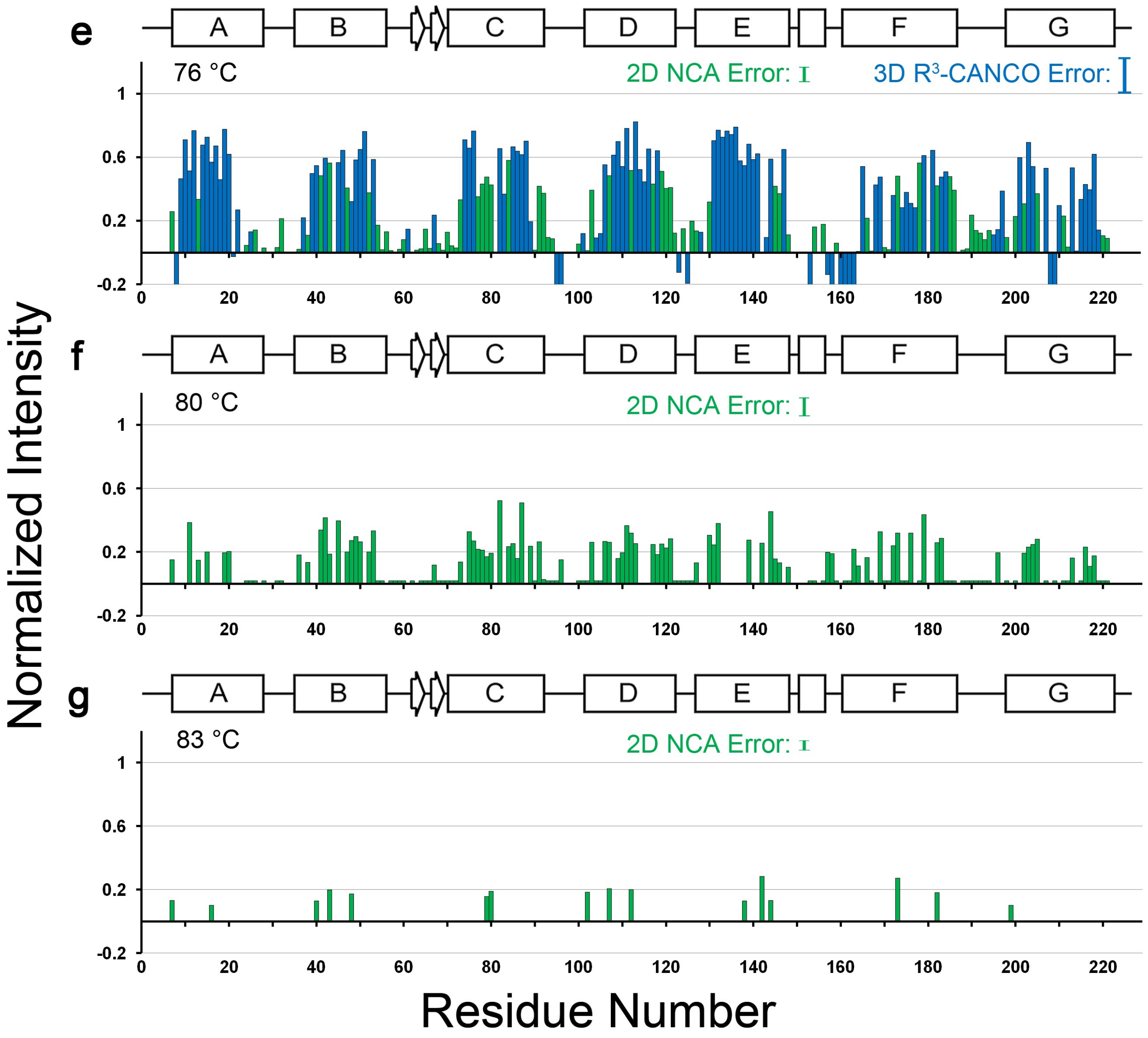

Supplementary Figure 5. Site-specific normalized cross-peak intensities as a function of temperature. Normalized intensities were obtained from the 3D R^3-CANCO (blue) and 2D NCA (green) spectra collected after the incubation in the D_2O based buffer at 20 °C (**a**), 48 °C (**b**), 60 °C (**c**), 68 °C (**d**), 76 °C (**e**), 80 °C (**f**) and 83 °C (**g**). Cross-peak intensities are normalized according to Equations (3-4). Negative intensities are due to the uncertainty in the R^3 scaling factor α. Only 2D NCA spectra were collected after incubation at 80 °C and 83 °C. All data were extracted from spectra collected on sample 1. Errors are estimated to be up to 10% in the 2D NCA and up to 20% in the 3D R^3-CANCO. Error bars are shown to guide the eye, and correspond to one standard deviation for the strongest peaks.

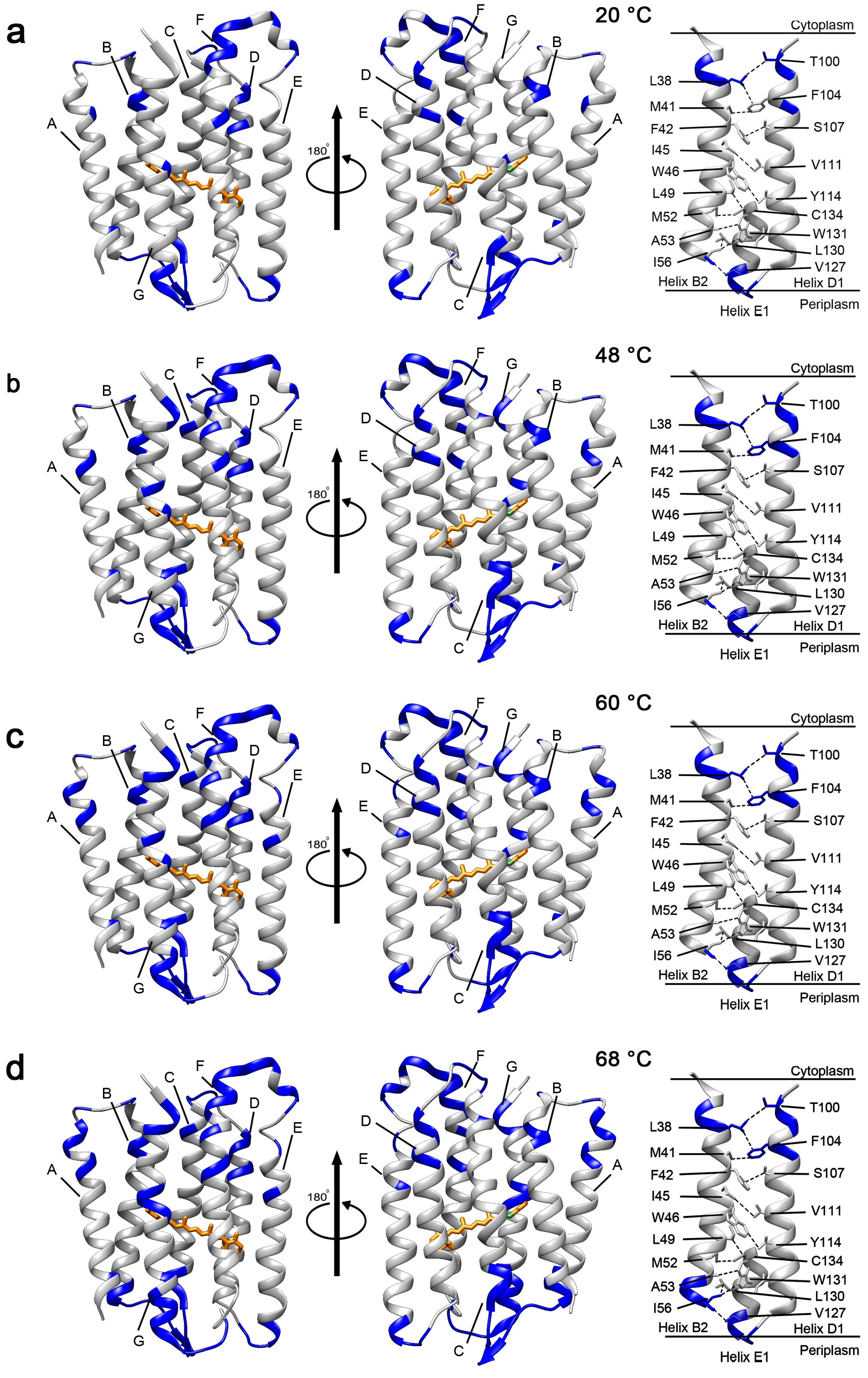
a
20 °C
b
48 °C
c
60 °C
d
68 °C
A
B
C
D
E
F
G
180°
Cytoplasm
Periplasm
L38
M41
F42
I45
W46
L49
M52
A53
I56
T100
F104
S107
V111
Y114
C134
W131
L130
V127
Helix B2
Helix D1
Helix E1

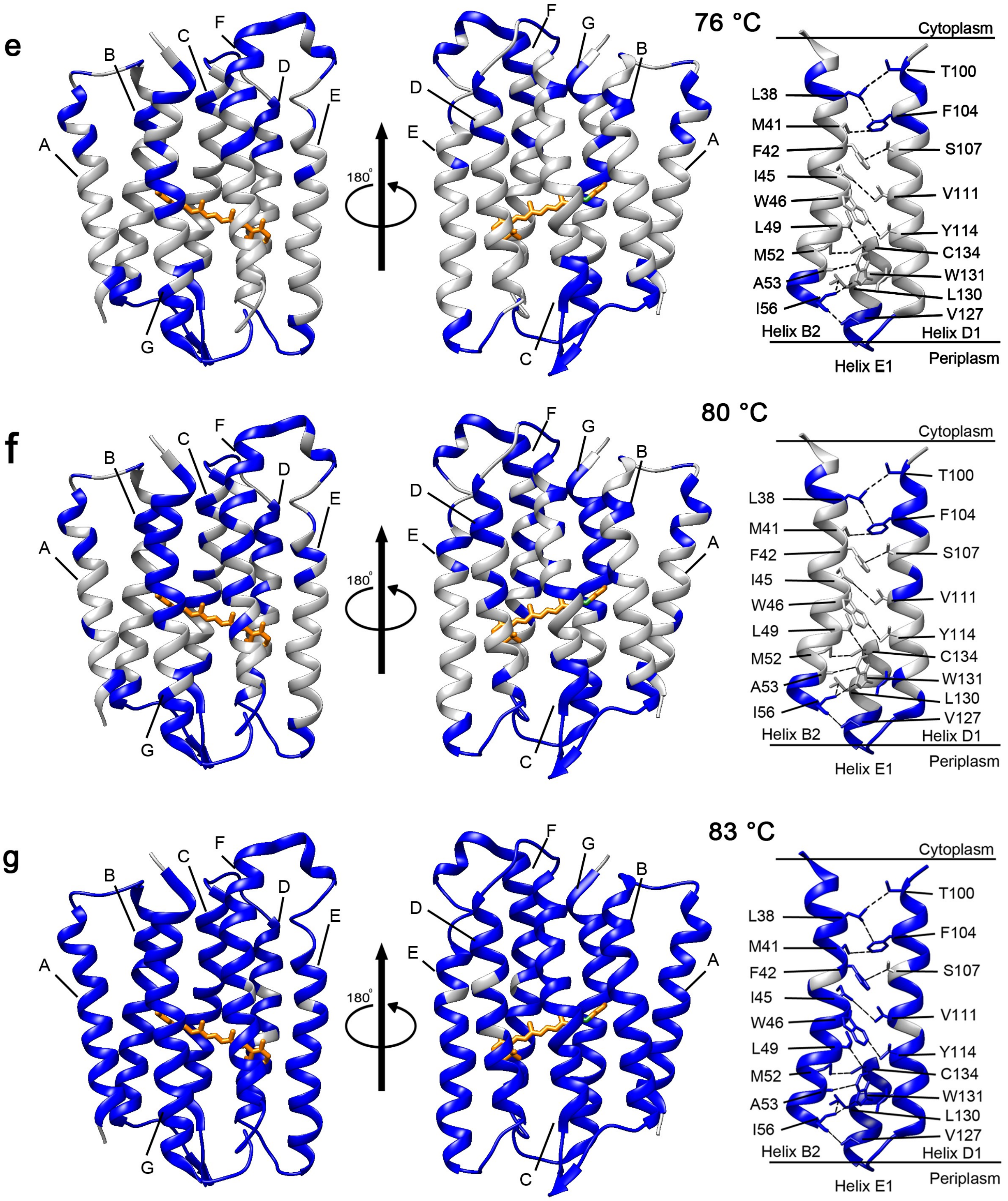

Supplementary Figure 6. Progression of H/D exchange as a function of temperature mapped on the ASR structure. (**a** to **g**) The structural models of ASR (PDB 5UK6) with exchange patterns at indicated temperatures. Exchanged sites are shown in blue, non-exchangeable sites in grey and retinal in orange. Monomers are shown on the left in each panel, and inter-monomer interface on the right. The exchange patterns were derived from experiments on sample 1 and confirmed through additional validation experiments on an independently prepared sample 2.

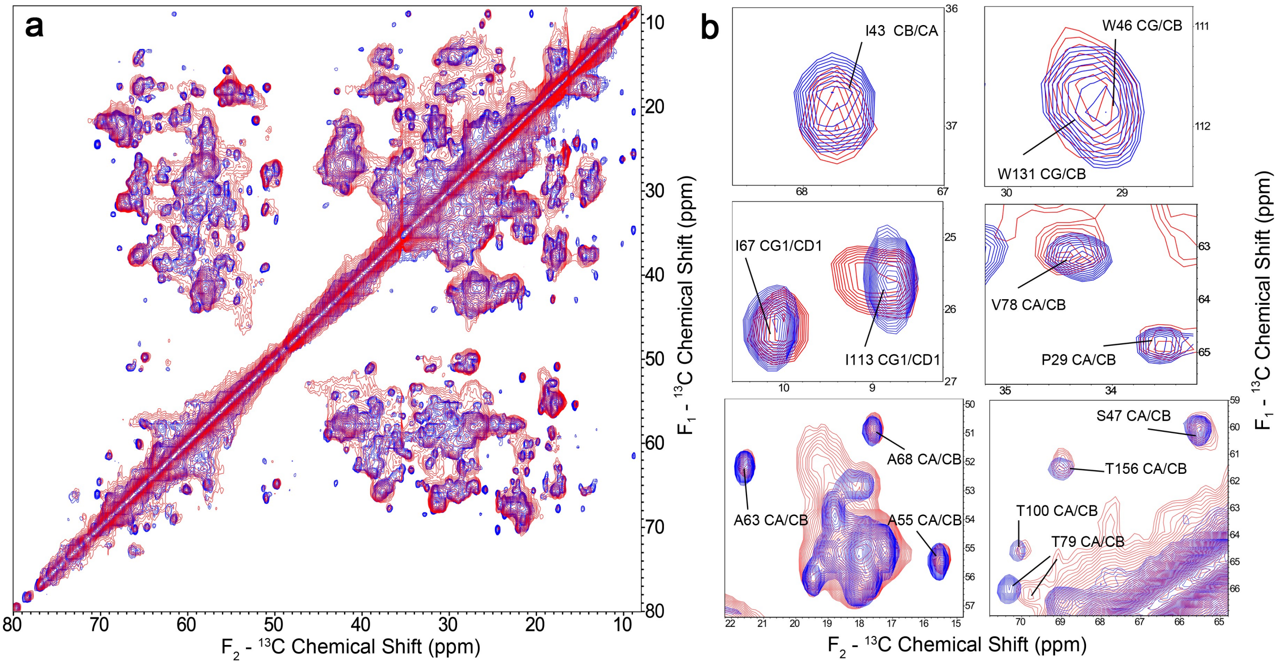

Supplementary Figure 7. 2D DARR ^{13}C-^{13}C correlation spectra collected on sample 1. Reference spectrum collected in the H_2O buffer before incubation is shown in blue, and the spectrum collected after the incubation in the D_2O buffer at 80 °C is in red. (**a**) The entire aliphatic region. (**b**) Highlights of the structurally conserved peaks that are neither shifted nor broadened. DARR mixing was 30 ms in both spectra.

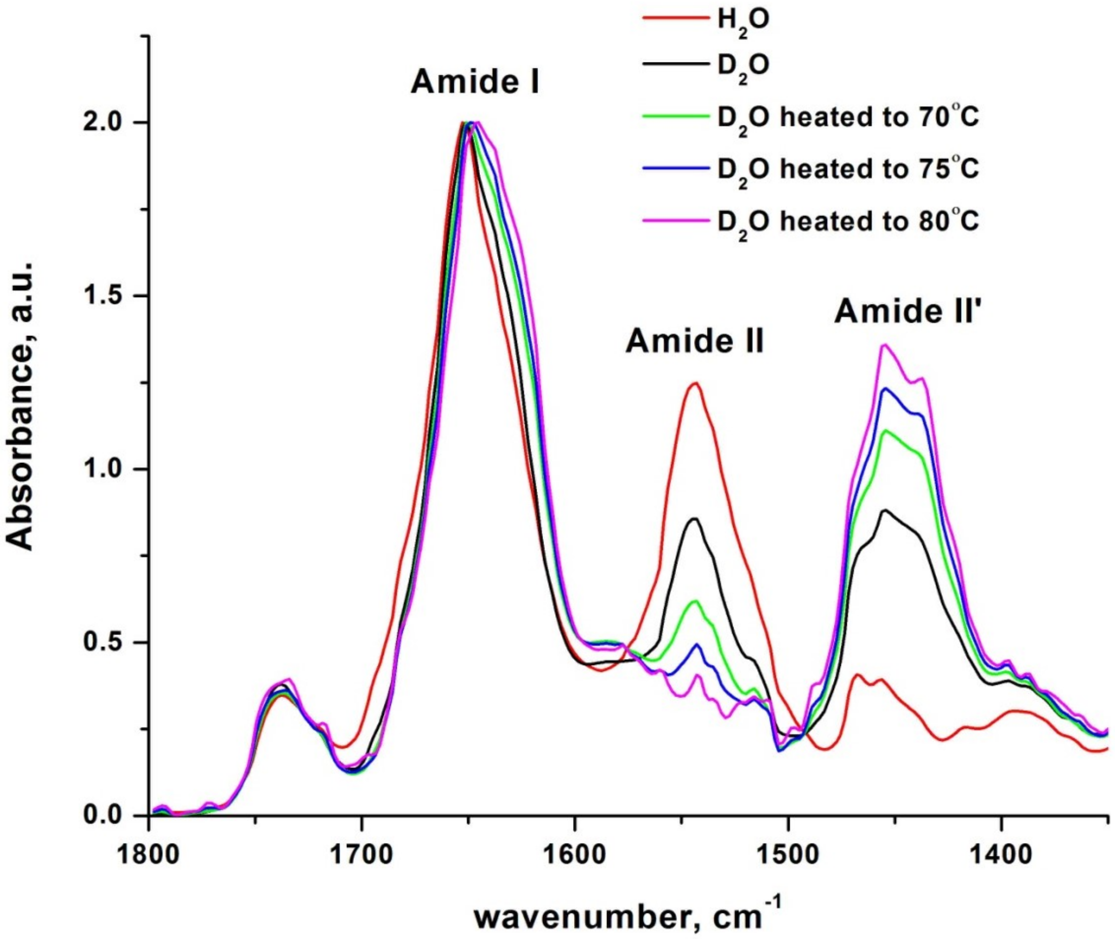

Supplementary Figure 8. H/D exchange and secondary structure changes during thermal unfolding of ASR in proteoliposomes observed by Attenuated Total Reflectance Fourier-Transform Infrared (ATR-FTIR) spectroscopy. The Amide II band represents protonated backbone nitrogen atoms, while the Amide II' band measures the fraction of the deuterated ones. The peak position of the Amide I (originally at 1653 cm^{-1}, typical for helical structures) reflects protein's secondary structure, where minor shifts (few cm^{-1}) reflect deuteration of the backbone, while larger shifts (such as the downshift to 1645 cm^{-1} and the appearance of a shoulder at 1630 cm^{-1}) represent partial loss of helicity upon heating. The spectra were normalized by the Amide I amplitude and taken at 30°C after heating to and incubating at a specified temperature as indicated in the figure. Additional details are given in the Methods.

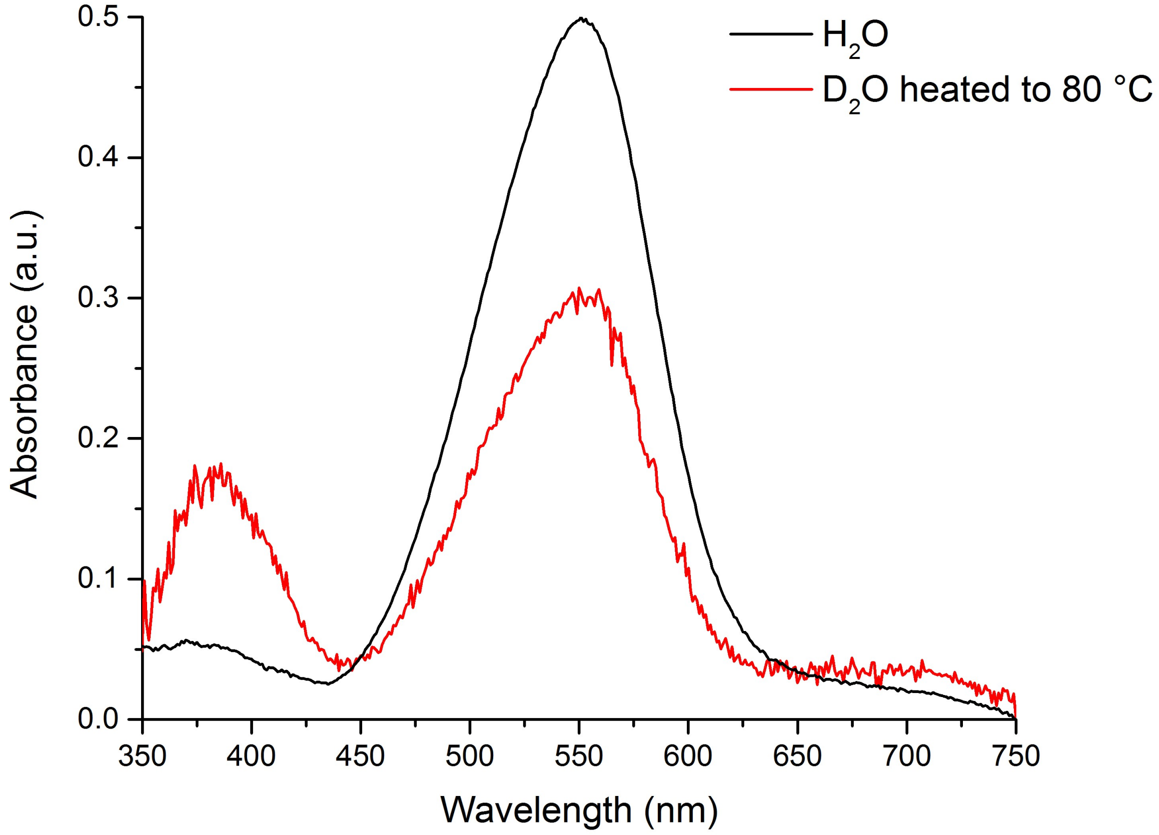

Supplementary Figure 9. The extent of irreversible loss of retinal in ASR proteoliposomes at 80 °C assayed by UV-Vis spectroscopy. Reference absorption spectrum collected at 20 °C prior to incubation is shown in black. Spectrum shown in red was collected at 20 °C after the incubation at 80 °C for 2 minutes. The peak at 550 nm corresponds to ASR-bound retinal and retains approximately 60 % of the initial intensity. The peak at ~370 nm corresponds to free retinal. The spectrum collected after the incubation at 80 °C was normalized according to Equation (2). Additional details are given in the Methods.

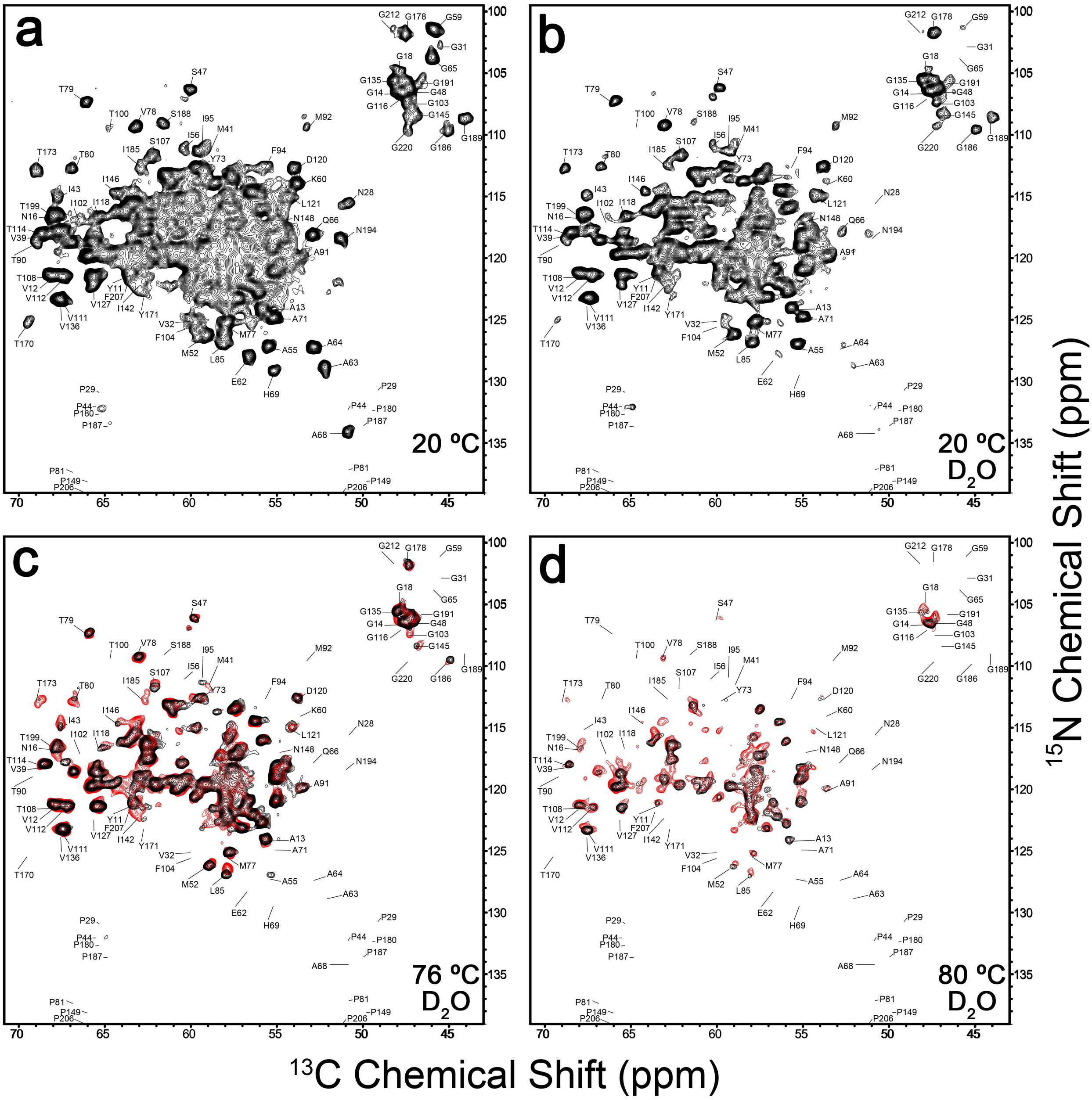

Supplementary Figure 10. A series of 2D NCA correlation spectra as a function of incubation temperature collected on independently prepared sample 2. (**a**) Reference 2D NCA spectrum collected in the H_2O based buffer. **(b)** 2D NCA spectrum collected after incubation in the D_2O based buffer at 20 °C. (**c** to **d**) Overlay of 2D NCA spectra collected on sample 1 (red) and sample 2 (black) after incubation at 76 °C (**c**), and at 80 °C (**d**).

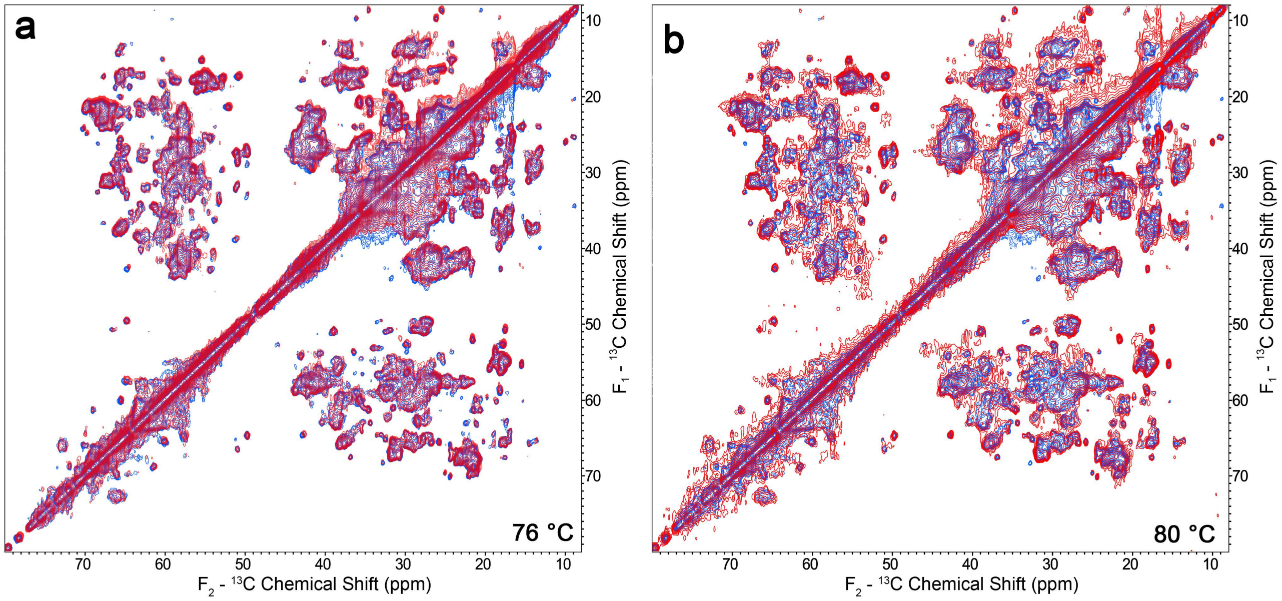

Supplementary Figure 11. 2D DARR ^{13}C-^{13}C correlation spectra collected on sample 2. (**a**) A comparison between the 2D ^{13}C-^{13}C correlation spectra collected before incubations (blue) and after incubation in D_2O at 76 °C (red). (**b**) A comparison between the 2D ^{13}C-^{13}C correlation spectra collected before incubations (blue) and after incubation in D_2O at 80 °C (red). DARR mixing was 30 ms in all experiments.

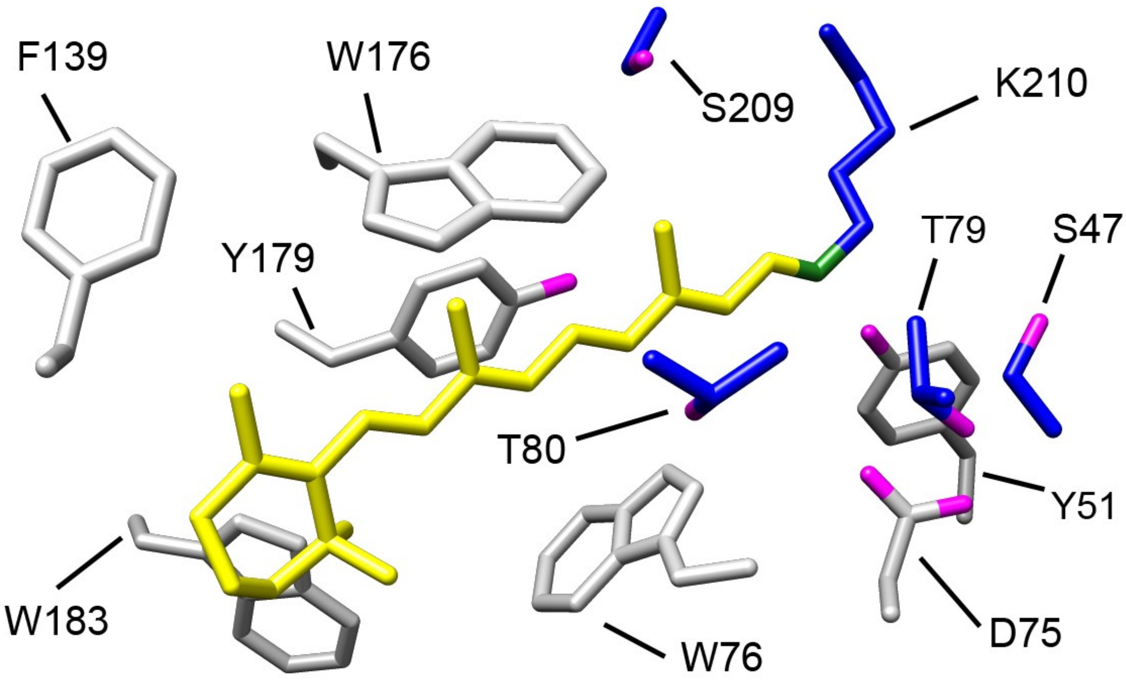

Supplementary Figure 12. Retinal-binding pocket of ASR (PDB 5UK6). Only the side chains of the residues are shown. The side chain oxygens are in magenta, and the Schiff base in green. Residues with exchanged amide sites after incubation at 80 °C are shown in blue, residues with non-exchanged amides are in white, and retinal in yellow.

Supplementary Table 1. Parameters of NMR experiments and analysis

**2D NCA**	
Acquisition length (t2/t1) (ms)	20/14.4
Total points (t2/t1)	2048/142
recycle delay (s)	1.5
number of scans	48 (sample 1)<br>440 (sample 2)
π/2 pulse ($^1H/^{13}C/^{15}N$) (μs)	2.5/5.0/7.0
CP contact time ($^1H/^{15}N$) (μs)	300
CP contact time ($^{15}N/^{13}C$) (ms)	6
Zero fill (t2/t1)	4096/1024
Lorentzian line narrowing ($^{15}N/^{13}C$) (Hz)	15/40
Gaussian line broadening ($^{15}N/^{13}C$) (Hz)	30/80
**3D R^3-CANCO**	
Acquisition length (t3/t2/t1) (ms)	20/9.5/6.3
Total points (t3/t2/t1)	2048/120/140
recycle delay (s)	1.5
number of scans	12
π/2 pulse ($^1H/^{13}C/^{15}N$) (μs)	2.5/5.0/7.0
CP contact time ($^1H/^{13}CA$) (ms)	2
CP contact time ($^{13}CA/^{15}N$) (ms)	6
CP contact time ($^{15}N/^{13}CO$) (ms)	6
CW power during R^3 recoupling (kHz)	14.3
R^3 recoupling period (μs)	839.16 (12 rotor cycles)
Zero fill (t3/t2/t1)	4096/1024/512
Lorentzian line narrowing ($^{15}N/^{13}C$) (Hz)	15/40
Gaussian line broadening ($^{15}N/^{13}C$) (Hz)	30/80
**2D ^{13}C - ^{13}C**	
Acquisition length (t2/t1) (ms)	22/7
Total points (t2/t1)	2612/1132
recycle delay (s)	1.4
number of scans	52 (sample 1)<br>104 (sample 2)
π/2 pulse ($^1H/^{13}C$) (μs)	2.5/5.0
CP contact time ($^1H/^{13}C$) (ms)	2
DARR mixing time (ms)	30
Zero fill (t2/t1)	4096/2048
Cosine bell function	
