# Supplementary material for: Solid-state NMR spectroscopy based atomistic view of a membrane protein unfolding pathway
Source: Nat Commun. 2019 Aug 27;10:3867. doi: 10.1038/s41467-019-11849-8 (PMC6711998; doi:10.1038/s41467-019-11849-8)
Supplement: Supplementary file 3 — Reporting Summary [file 41467_2019_11849_MOESM3_ESM.pdf]

nature research

Corresponding author(s): Leonid S. Brown, Vladimir Ladizhansky

Last updated by author(s): May 15, 2019

Reporting Summary

Nature Research wishes to improve the reproducibility of the work that we publish. This form provides structure for consistency and transparency in reporting. For further information on Nature Research policies, see Authors & Referees and the Editorial Policy Checklist.

Statistics

For all statistical analyses, confirm that the following items are present in the figure legend, table legend, main text, or Methods section.

n/a	Confirmed	
☒	☐	The exact sample size (*n*) for each experimental group/condition, given as a discrete number and unit of measurement
☐	☒	A statement on whether measurements were taken from distinct samples or whether the same sample was measured repeatedly
☒	☐	The statistical test(s) used AND whether they are one- or two-sided<br>*Only common tests should be described solely by name; describe more complex techniques in the Methods section.*
☒	☐	A description of all covariates tested
☒	☐	A description of any assumptions or corrections, such as tests of normality and adjustment for multiple comparisons
☒	☐	A full description of the statistical parameters including central tendency (e.g. means) or other basic estimates (e.g. regression coefficient) AND variation (e.g. standard deviation) or associated estimates of uncertainty (e.g. confidence intervals)
☒	☐	For null hypothesis testing, the test statistic (e.g. *F*, *t*, *r*) with confidence intervals, effect sizes, degrees of freedom and *P* value noted<br>*Give P values as exact values whenever suitable.*
☒	☐	For Bayesian analysis, information on the choice of priors and Markov chain Monte Carlo settings
☒	☐	For hierarchical and complex designs, identification of the appropriate level for tests and full reporting of outcomes
☒	☐	Estimates of effect sizes (e.g. Cohen's *d*, Pearson's *r*), indicating how they were calculated

Our web collection on statistics for biologists contains articles on many of the points above.

Software and code

Policy information about availability of computer code

Data collection	NMR data were collected on Bruker NMR spectrometer using Bruker Topspin software, version 3.2.<br>Differential Scanning Calorimetry (DSC) experiment was performed on a TA Instruments Nano DSC 602000.901 unit using DSCRun 4.2.6.<br>UV-Vis spectroscopy was performed on a Varian Cary-50 spectrometer, operating on Cary WinUV Scan Application version 3.0.<br>FTIR measurements were conducted using a temperature-controlled Germanium Attenuated Total Reflectance (ATR) accessory (Pike Technologies, Madison WI) installed in a Bruker Vertex 70 FTIR spectrometer, operating on OPUS version 7.5.
Data analysis	NMR spectra were processed using NMRpipe (https://www.ibbr.umd.edu/nmrpipe/index.html). Cross peak intensities were analyzed using Computer Aided Resonance Assignments (CARA) software, version 1.8.4.2 (http://cara.nmr-software.org/portal/).<br>Structure visualization was done using UCSF Chimera software version 1.13.1 (https://www.cgl.ucsf.edu/chimera/).<br>DSC data were analyzed using TA Instruments NanoAnalyze software version 3.7.5.<br>FTIR data were analyzed using Bruker OPUS software version 7.5 and Origin version 7.0.

For manuscripts utilizing custom algorithms or software that are central to the research but not yet described in published literature, software must be made available to editors/reviewers. We strongly encourage code deposition in a community repository (e.g. GitHub). See the Nature Research guidelines for submitting code & software for further information.

Data

Policy information about availability of data

All manuscripts must include a data availability statement. This statement should provide the following information, where applicable:

- Accession codes, unique identifiers, or web links for publicly available datasets
- A list of figures that have associated raw data
- A description of any restrictions on data availability

The data sets generated and analysed during the current study are available from the corresponding author on reasonable request.

Field-specific reporting

Please select the one below that is the best fit for your research. If you are not sure, read the appropriate sections before making your selection.

☒ Life sciences ☐ Behavioural & social sciences ☐ Ecological, evolutionary & environmental sciences

For a reference copy of the document with all sections, see nature.com/documents/nr-reporting-summary-flat.pdf

Life sciences study design

All studies must disclose on these points even when the disclosure is negative.

Sample size	Two independently prepared protein samples were used in this study. Full data set consisting of 2D NCA, 3D R-3CANCO was collected on sample 1. Validation data set consisting of 2D NCA and 2D carbon-carbon correlation experiments was collected on sample 2. Statistical analysis and error estimation was based on the analysis of NMR spectral noise.
Data exclusions	No data were excluded
Replication	Experiments were carried out under clearly defined conditions. On sample 1 two-dimensional NCA experiments were carried out twice for each temperature point where applicable, to ensure sample stability and data over the time course of three-dimensional NMR experiments.<br>All replication experiments were successful.<br>DSC data were successfully reproduced twice.<br>FTIR data were replicated successfully.
Randomization	Not applicable to this study
Blinding	Not applicable to this study

Reporting for specific materials, systems and methods

We require information from authors about some types of materials, experimental systems and methods used in many studies. Here, indicate whether each material, system or method listed is relevant to your study. If you are not sure if a list item applies to your research, read the appropriate section before selecting a response.

Materials & experimental systems

n/a	Involved in the study	
☒	☐	Antibodies
☒	☐	Eukaryotic cell lines
☒	☐	Palaeontology
☒	☐	Animals and other organisms
☒	☐	Human research participants
☒	☐	Clinical data

Methods

n/a	Involved in the study	
☒	☐	ChIP-seq
☒	☐	Flow cytometry
☒	☐	MRI-based neuroimaging
